# On Arterial Compression in Inflammation of the Extremities

**Published:** 1849-06

**Authors:** Francois Henroz


*On Arterial Compression in Inflammation of the Extremities.* By Dr. Francois Henroz.—Dr. Henroz suggests the employment of arterial compression in the treatment of the different inflammatory affections, both of the superior and inferior extremities.

He himself has only made use of it in two cases of paronychia, in which it gave complete and instantaneous relief from pain, and in a short time caused resolution of the inflammation. In severe cases, the pressure ought to be applied either upon the humeral artery, or upon both the arteries of the fore-arm; but in general, pressure upon the radial, when the thumb, or index, or middle finger is affected; or of the cubital, when the ring or little finger is the seat of the disease, will be found sufficient. The apparatus which he employs is exceedingly simple; it consists merely of two small wooden splints, somewhat longer than the diameter of the limb at the part to be compressed. A small compress having been placed over the artery, one of the splints is applied upon it, and the other opposite to it, and their ends are tied together beyond the limb on either side. The amount of pressure can thus be easily regulated.

In one of the cases in which he employed this treatment, the disease had been going on for eight days before the patient was seen by him. All the fingers were affected, and the whole of the hand and lower part of the fore-arm were very much inflamed, but no fluctuation could be detected. The axillary glands had become swollen and painful, and there was general feverish reaction. Pressure with the hand over the humeral artery immediately caused a cessation of the pain. The apparatus was applied so as to compress both the arteries of the fore-arm above the wrist, and no bad effects were experienced, notwithstanding the inflamed state of the parts.

The splints were directed to be removed for a short time every three or four hours. No pain was felt as long as the pressure was continued, but when it was removed the pain returned. Next morning the constitutional disturbance had quite subsided, the swelling and heat of the hand were much diminished: and after the pressure had been continued about thirty hours, all the symptoms of inflammation had disappeared. *Bulletin de l'Academie Royale de Médecine de Belgique.*

[It seems doubtful how far the relief from pain obtained in those cases was the result of compression of the arteries, and not rather of pressure upon the nerves, as we know that the phenomenon of pain generally precedes the increased flow of blood to an inflamed part. It seems also very doubtful whether compression may not sometimes produce such effects as shall render its use improper. Nevertheless, we have been induced to give this abstract of M. Henroz's memoir, from the belief that the subject merits further investigation.—*London Monthly Journal.*]

